# Neutrophil-to-Lymphocyte Ratio as Potential Marker of Outcome in Popliteal Artery Aneurysm Repair

**DOI:** 10.3390/biomedicines13030651

**Published:** 2025-03-06

**Authors:** Pasqualino Sirignano, Elisa Romano, Giulia Colonna, Flavia Del Porto, Costanza Margheritini, Chiara Pranteda, Nazzareno Stella, Maurizio Taurino, Luigi Rizzo

**Affiliations:** 1 Vascular and Endovascular Surgery Unit, Sant’Andrea Hospital of Rome, Department of General and Specialistic Surgery, “Sapienza” University of Rome, 00189 Rome, Italy; pasqualino.sirignano@uniroma1.it; 2 Vascular and Endovascular Surgery Unit, Sant’Andrea Hospital of Rome, Department of Molecular and Clinical Medicine, “Sapienza” University of Rome, 00189 Rome, Italy; giulia.colonna@uniroma1.it (G.C.); c.margheritini@gmail.com (C.M.); cpranteda@ospedalesantandrea.it (C.P.); nstella@ospedalesantandrea.it (N.S.); maurizio.taurino@uniroma1.it (M.T.); luigi.rizzo@uniroma1.it (L.R.); 3 Internal Medicine Unit, Sant’Andrea Hospital of Rome, Department of Molecular and Clinical Medicine, “Sapienza” University of Rome, 00189 Rome, Italy; flavia.delporto@uniroma1.it

**Keywords:** popliteal artery aneurysm, neutrophil to lymphocyte ratio, inflammation, vascular outcomes

## Abstract

**Objective:** The neutrophil–lymphocyte ratio (NLR) is an inexpensive and easily available inflammatory marker for cardiovascular disease. The aim of the present study is to evaluate a possible association between preoperative NLR value and popliteal artery aneurysm (PAA) repair outcomes. **Methods:** A single-center retrospective study on all patients, who underwent urgent or elective PAA repair from June 2010 to October 2022, was performed. Study outcomes were immediate technical success, 30-day and mid-term primary patency, reintervention, limb salvage, and mortality rates. The NLR was calculated by dividing the absolute neutrophil count by the absolute lymphocyte count, and, according to the literature, a cut-off of five has been considered as a possible threshold for the analysis. **Results:** Eighty-two patients (80 male) with a total of 97 popliteal artery aneurysms were enrolled in this study. The mean preoperative NLR was 2.9 ± 2.4. In 10 (10.3%) PAAs, the NRL was >5 (High-NLR group), and, in the remaining 87 (89.7%), the NLR was <5 (Low-NLR group). The preoperative NLR for urgent procedures was higher than elective cases (4.37 vs. 2.30; *p* < 0.001). However, no significant differences were found as far as immediate 24 h technical success (*p* = 0.48) and 30-day primary patency (*p* = 39). At mean follow-up, the primary patency rate was significantly higher in the Low-NLR group (*p* = 0.0044), without statistical differences for re-operation (*p* = 0.27), limb salvage (*p* = 0.09), and mortality rates (*p* = 0.51). The Kaplan–Mayer analysis showed a significant difference in freedom from major amputation in patients with an NLR > 5 compared to the ones with an NLR < 5 (*p* = 0.038), without any differences in terms of survival, primary patency, and the need for reintervention rates. The multivariable Cox regression analysis identified the NLR value as an independent predictor of better outcomes regarding freedom from the amputation rate (*p* = 0.25). **Conclusions:** Our experience indicates that a preoperative NLR value > 5 can identify high-risk patients affected by a PAA and may negatively influence the surgery’s long-term outcomes. Therefore, this selected group of patients could need a more tailored approach and closer monitoring over time.

## 1. Introduction

Although rare, with an estimated incidence of 0.1–2.8%, popliteal artery aneurysms (PAAs) are the most common peripheral arterial aneurysms, accounting for 70% of all aneurysms [1]. PAAs are usually diagnosed in asymptomatic or mild symptomatic (intermittent claudication, IC) patients, but they could be complicated by acute thrombosis and acute limb ischemia (ALI), distal embolism with the subsequent risk of critical limb ischemia (CLI), and, rarely, rupture [2,3,4].

The Society for Vascular Surgery (SVS) guidelines recommend treatment for PAAs > 20 mm for asymptomatic patients and for PAAs < 20 mm in case of the presence of thrombus and the clinical suspicion of embolism or the imaging evidence of poor distal runoff to prevent thromboembolic complications and potential limb loss [2].

Despite the different kinds of surgical or endovascular treatments, the amputation rate in patients affected by popliteal artery aneurysms remains high, especially in patients with acute presentation [5,6].

Consequently, the need for new and different prognostic values has emerged. The neutrophil-to-lymphocyte ratio (NLR; normal range 1–3) is an easy test derived from the white blood cell count and reflects the balance of the neutrophilia of inflammation and the relative lymphopenia of a cortisol-induced stress response [7,8]. The NLR has already been tested and proven as a strong morbidity or mortality predictor in several acute and chronic cardiovascular diseases, such as atrial fibrillation, heart failure, ALI, aortic dissections, and aneurysms, but its role in peripheral aneurysms is still under investigation [7,8,9,10].

The aim of this study is to investigate the role of preoperative NLR in predicting clinical outcomes in an unselected population presenting with both symptomatic and asymptomatic PAAA who underwent surgical or endovascular intervention in a referral tertiary Italian academic hospital.

## 2. Materials and Methods

A single-center retrospective study was performed between June 2010 and October 2022 on all consecutive PAA patients surgically treated at the Vascular and Endovascular Surgery Unit of a tertiary referral academic hospital (Sant’Andrea Hospital—Sapienza University of Rome). According to the latest SVS guidelines, the indications for treatment were diameter >20 mm, presence of PAA-associated symptoms as intermittent claudication (IC), chronic limb threatened ischemia (CLTI), or acute limb ischemia (ALI), and rupture [2]. Patients were categorized as urgent or elective cases according to their clinical presentation: asymptomatic patients or those presenting solely with IC were managed electively, whereas all other conditions warranted an urgent approach. Patients with a diagnosis of PAA but without surgical or endovascular indication and those refusing treatment were excluded. Patients affected by any kind of neoplasia, ongoing infection, or autoimmune disorder were excluded since these conditions have been proven to elevate NLR values [11]. For each patient included in this study, a single pre-operative blood sample was collected to measure NLR values. Each limb was treated independently: this approach reflects the fact that the progression of disease on each side in the same patient can vary significantly and is not necessarily interconnected. Indeed, for each patient with bilateral disease, we determined a different pre-operative NLR for both PAAs. Moreover, other studies in the literature have used this method to analyze bilateral diseases [12]. The NLR was calculated as the ratio of neutrophil-to-lymphocyte counts and was subsequently used in all analyses. Blood counts were collected in test tubes with anticoagulant DTA-K3 and processed through cytometric analysis (Alinity h-series^®^, Abbott Laboratories, Des Plaines, IL, USA).

### 2.1. Preoperative Work-Out

All patients underwent an extensive preoperative assessment, including clinical history reporting, physical examination, chest radiography, electrocardiography, transthoracic echocardiography, and laboratory testing. Duplex ultrasound (DUS) and computed tomographic angiography (CTA) were performed in all cases. CTAs were performed with and without contrast medium during arterial and venous phases, at a thickness of 1 mm. All measurements (diameter, length, angles) were evaluated using a workstation with dedicated reconstruction software (OsiriX^®^ MD software version 12, PIXMEO, Bernex, Switzerland, and Horos Open Software^®^ version 4 on Mac OS compute) [13]. High-skilled vascular surgeons evaluated all examinations for each patient. Digital Subtraction Angiography (DSA) was never selected as diagnostic tool, while it was routinely adopted as intraoperative adjunct in case of loco-regional transcatheter fibrinolytic therapy.

### 2.2. Surgical and Endovascular Procedures

According to SVS guidelines [2], therapeutic approach was chosen based on symptoms at presentation, PAA anatomical characteristics, patient’s general status, and surgeon’s choice. All interventions were performed by vascular surgeons, except for the fibrinolysis that was performed together by vascular surgeons and interventional radiologists.

The surgical technique was chosen on a patient-by-patient basis. In case of open repair (OR), a posterior approach was chosen in short aneurysms limited to the popliteal fossa, whereas a medial approach was preferred for lesions extending above and below the knee joint. Several conduits were used for arterial reconstruction according to the characteristics of the aneurysm and of the inflow and outflow vessel status. In general, a polytetrafluoroethylene (PTFE) prosthetic graft was chosen in patients with aneurysms not involving the distal vessels and with a good runoff status or when a good quality ipsilateral saphenous vein was lacking, while a great saphenous vein (GSV) bypass was preferred in patients with long lesions extending well below the knee or with a poor run-off. Endovascular procedures (EVs) were performed in an operating room equipped with portable fluoroscopy unit (OEC 9900 Elite^®^; General Electric Company; Boston, MA, USA). All procedures were performed through an ipsilateral or contralateral access at the common femoral artery level. The choice between surgical cutdown or percutaneous technique was derived according to introducer sheath diameter: percutaneous access was considered for diameters ranging from 7 to 9F, while surgical access was used for greater diameters up to 11F. After percutaneous approach, hemostasis was achieved by 45 min of manual compression or dedicated devices: Angioseal^®^ (St. Jude Medical, St. Paul, MN, USA) and FemoSeal^®^ (St. Jude Medical). The Viabahn^®^ peripheral endograft (W. L. Gore and Associates, Inc., Flagstaff, AZ, USA) was used in all cases. Viabahn diameter was determined by the preoperative CTA and DUS imaging considering a 10% to 15% oversizing respect to luminal diameter. Proximal and distal landing zones were required to be at least 15 mm in length. When more than 1 stent graft was needed, the deployment was performed in caudal-cranial fashion, with a minimum overlapping of at least 20 mm between the grafts. Post-dilatation was performed by a noncompliant balloon at landing zones and overlapping sites as well. A completion angiogram was performed with and without knee flexion to evaluate runoff, kinks, or occlusions caused by the bending of the knee.

In case of PAA presenting with either thrombotic or embolic ALI without visible outflow vessels, preoperative catheter-directed thrombolysis was administered before definitive OR or EV treatment [7].

### 2.3. Follow-Up Protocol

Follow-up protocol consisted of clinical and DUS examinations at 1, 6, and 12 months post-intervention, and yearly thereafter. In the absence of clinical complications, CTA was never performed during follow-up.

### 2.4. Study Outcomes

Early (30 days) results were analyzed for mortality, graft thrombosis and reintervention rates. Follow-up results, including survival, primary and secondary patency, freedom from amputation, and freedom from reintervention, were also analyzed. Primary patency was defined as uninterrupted patency without additional procedures at the margin of the treated segment, whereas secondary patency was defined as restored patency through the originally treated segment.

### 2.5. Ethical Approval

This study was conducted according to the guidelines of the Declaration of Helsinki and approved by the Local Ethics Committee of “Sapienza” University of Rome—Policlinico Umberto I Hospital and Sant’Andrea Hospital (Project dentification code 384/17, date of approval: 3 March 2017). All patients enrolled in this study gave their informed written consent to be submitted for intervention and to be included in the present analysis.

### 2.6. Statistical Analysis

Preoperative demographic and clinical characteristics, NLR values, and technical features were evaluated as independent factors potentially influencing the outcome. Enrolled patients were categorized into High- and Low-NLR groups in accordance with their preoperative NLR: a cut-off of 5.00 was considered as a possible threshold for the analysis, according to previously published studies performed in different fields of vascular diseases [7,14]. Demographic and clinical data were displayed as mean (±SD) for normally distributed data, as median (25th to 75th percentile) for non-parametric data, and as frequencies (percentages) for the categorical data. The distribution of continuous data was tested using Kolmogorov–Smirnov tests. Categorical data were compared using Fisher exact test, and continuous variables were compared using independent sample T-test or Mann–Whitney U test based on the type of distribution. Odds ratio and risk ratio were calculated to study the primary endpoint for clinical and procedural variables. Freedom from adverse event occurrence estimation was based on the cumulative incidence function according to the Kaplan–Meier method. Multivariate Cox proportional hazard models were used to examine mortality, primary patency, reintervention, and freedom from major amputation for adjusted associations with demographic parameters at mean time follow-up. Resulting hazard ratios and 95% confidence intervals were calculated for each covariate in the multivariate analysis.

A 2-sided value of *p* < 0.05 was considered statistically significant. Statistical tests were performed using SPSS 27.0 (IBM Corp, Armonk, NY, USA, 2020).

## 3. Results

Ninety-seven PAAs were detected in 82 patients during the entire study period. Eighty patients (97.6%) were males, and thea mean age of the cohort was 69.3 ± 9.3 years (range 49–90). The mean popliteal aneurysm diameter was 31.9 ± 5.7 (range 13–97); a bilateral PAA was detected in 32 patients (39%), and, in 26 (26.8%), a concurrent abdominal aortic aneurysm was diagnosed. The mean preoperative NLR value was 2.9 ± 2.4 (range 0.8–18.7). Ten cases (10.3%) presented with an NLR value > 5.00 (High-NLR group) and the remaining 87 (89.7%) with an NLR < 5.00 (Low-NLR). Demographic and clinical characteristics of all patients included in the present series are reported in Table 1.

Thirty cases (30.9%) required an urgent treatment (one for PAA rupture), while the great majority of PAAs were preventively treated in elective fashion. Seventy-seven (79.3%) PAAs underwent OR and 20 an EV (20.6%); in 16 cases (16.5%), a preoperative fibrinolytic treatment was additionally performed before definitive treatment (mean duration 40.4 ± 22.5 h; range 8–96).

Immediate technical success was obtained in 93 cases (95.9%), without significant differences between the OR and EV (94.8% vs. 100%; *p* = 0.31; OR1.04: CI95%: 0.10–9.86), or urgent and elective procedures (93.3% vs. 97%; *p* = 0.39; OR: 2.32 CI95%: 0.31–17.31). Details of performed reintervention are reported in Table 2.

All patients completed the 30-day follow-up, and the mortality rate was null. Primary patency was maintained in 93 cases (95.8%) without differences between elective and urgent (3/30) patients (*p* = 0.051; OR: 0.13 CI95%: 0.01–1.36), or surgical (3/77) and endovascular (1/20) interventions (*p* = 0.82; OR: 0.77 CI95%: 0.07–7.82). Eight reinterventions were performed; details are reported in Table 2.

At a mean-time follow-up of 46.54 months (range 1–144), the primary patency rate was 80.95%, the freedom from reintervention was 76.2%, limb salvage and mortality rates were 92.86%, and 30.95%, respectively. Electively treated patients presented significantly better results in all outcomes (primary patency *p* = 0.008; OR: 3.76 CI95%: 1.35–10.43; Freedom from reintervention *p* = 0.012; OR: 3.66 CI95%: 1.28–10.44; Freedom from amputation *p* = 0.005; OR: 12.72 CI95%: 1.4–115.20; Mortality *p* = 0.018; OR: 3.14 CI95%: 1.18–8.34).

Looking at the NLR value as an independent outcome variable, the mean preoperative value was found significantly higher in urgent cases compared to electively performed ones, 4.37 versus 2.30 (*p* < 0.00001). However, no significant differences were found as far as immediate 24 h technical success (*p* = 0.48; OR: 0.21 CI95%: 0.001–114.16) and 30-day primary patency (*p* = 0.39; OR: 0.13 CI95%: 0.001–72.42), between patients with high or low NLR preoperative values.

At univariate analysis, the cumulative primary patency rate was significantly higher in the Low-NLR group (*p* = 0.0044; OR: 0.15 CI95%: 0.034–0.63), although, no statistical differences in terms of reintervention (*p* = 0.27; OR: 0.32 CI95%: 0.03–2.71), limb salvage (*p* = 0.09; OR: 0.22 CI95%: 0.03–1.45) and mortality rates (*p* = 0.51; OR: 1.57 CI95%: 0.40–6.14) were determined at mean time follow-up.

**Figure 1:**
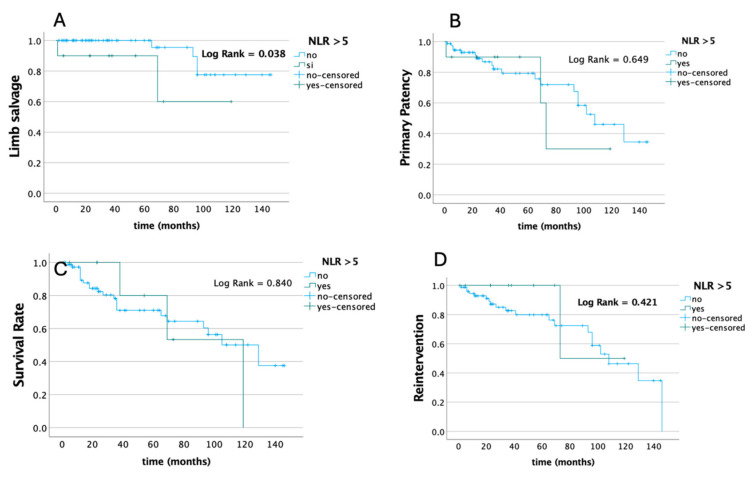
Estimated long-term freedom from any adverse events evaluated by Kaplan–Mayer curve analysis and log-rank test according to baseline NLR value in terms of primary patency (**A**), survival (**B**), freedom from major amputations (**C**), and need for reintervention rates (**D**).

Conversely, estimated long-term freedom from any adverse events evaluated by the Kaplan–Mayer curve analysis and log-rank test according to the preoperative NLR value showed a significant difference in terms of freedom from major amputation on the base of the preoperative NLR value (*p* = 0.038), without any differences in terms of survival, primary patency, and the need for reintervention rates (Figure 1).

The multivariable Cox regression analysis of mean time follow-up confirmed a significant correlation between NLR values and freedom from major amputation (*p* = 0.25; HR 0.033 CI95%: 0.002–0.652) (Table 3).

However, the multivariable Cox regression analysis of mean time follow-up did not show any significant association between NLR values and all other outcomes (primary patency, reintervention, and mortality), as an independent variable. More details are shown in Table 4, Table 5 and Table 6.

## 4. Discussion

The present study analyses the predictive value of preoperative NLR in defining clinical outcomes among patients affected by asymptomatic and symptomatic PAA, who underwent surgical or endovascular treatment. To the best of our knowledge, this is the first study conducted on the role of the NLR in PAAs.

Elective cases, as expected, demonstrated better outcomes at mean follow-up, highlighting the importance of screening and early therapeutic intervention.

It is well established in the literature that atherosclerotic disease and chronic inflammation are tightly connected. During chronic inflammation, neutrophils are constantly drawn to the site, contributing to tissue damage and intensifying the immune response. In contrast, lymphocytes are thought to play a protective role during inflammatory stages [14]. The NLR, expressing the ratio between neutrophils and lymphocytes, is typically elevated in inflammatory conditions and could be a useful marker for risk stratification in cardiovascular disease [4,15]. Previous studies demonstrated that neutrophils contribute to coagulation and atherosclerosis by promoting thrombin generation through neutrophil extracellular traps (NETs), which in turn activate platelets and trigger ischemic events [15]. Previous studies have shown the prognostic significance of the preoperative NLR value in vascular diseases, highlighting the need for a standardized cut-off value to identify high-risk patients [7,8]. Tokgoz et al. demonstrated that an NLR higher than 5.67 was a strong predictor of mortality after acute ischemic stroke, with a sensitivity of 81.7% and a specificity of 65.8% [16]. Likewise, the value of a preoperative NLR >5 has proven to be a valuable independent predictor of 30-day mortality in patients treated for abdominal aortic aneurysms (AAAs) [17]. Pasqui et al.^.^ reported a strong association between a preoperative NLR and the amputation rate in patients with acute limb ischemia (ALI), identifying the value of an NLR over 6.66 as an independent risk factor [12]. Our center previously investigated the relationship between the NLR and adverse event occurrence after treatment in unselected ALI patients, finding a significant difference between patients with high (>5) or low (<5) NLR values [4]. Based on these previous findings, an NLR cut-off value of 5 was selected in the present study.

Our study revealed that elective patients had a significantly lower NLR value than urgent cases, confirming that the NLR, as a marker of atherosclerotic inflammation, is strongly associated with the severity of the clinical condition. The NLR may therefore help identify selected groups of patients at increased risk of highly symptomatic PAA and could potentially lead the therapeutic management and the timing of surgery.

While no significant difference was found between high (>5) and low (<5) NLR groups of patients regarding immediate technical success and 30-day primary patency, patients with a low NLR had a significantly higher primary patency at meantime follow-up and a higher time of freedom from major amputation at a Kaplan–Mayer analysis. This last result was confirmed at multivariate Cox regression analysis, demonstrating that the NLR can be an independent predictor of limb salvage at mean time follow-up despite the presence/absence of other comorbidities.

The NLR reflects the overall clinical condition of patients, and it is strongly associated with long-term outcomes rather than immediate surgical ones, primarily dependent on the technical aspects of the procedure. Some authors recently demonstrated that the NLR, as an indicator of the systemic inflammatory grade (SIG), is linearly correlated with increased long-term mortality rates after EVAR [18]. A comparable mechanism can be supposed in popliteal aneurysms.

From a speculative point of view, the NLR could be used to select high-risk patients who are more susceptible to long-term complications and require careful monitoring during follow-up, as also suggested by the previous literature [7,11].

This study is not free from limitations, primarily due to its retrospective design. First, our analysis did not account for pre-operative and post-operative pharmacological therapy, which may have introduced some bias. Moreover, the study cohort is small, and the number of patients with a high NLR is poor (n = 10) compared to those with a low NLR (n = 87). Hence, the statistical power and significance of the results should be considered with caution. Second, since an elevated NLR is associated with various cardiovascular diseases, it may lack the specificity to identify patients with high-risk PAA. Additional biomarkers may be required to supplement the NLR in risk stratification and should be included in further research. Lastly, due to the retrospective design of this study, data on differences in functional outcomes and quality of life between patients in the Low and High-NLR groups were unavailable. Future prospective studies could address this limitation. Further large-scale clinical trials and a multivariate analysis should be conducted to validate these results and establish the NLR as an independent marker of high-risk patients affected by PAA in routine clinical practice.

## 5. Conclusions

Our experience, although limited, demonstrated that the NLR plays a key role in the preoperative selection of critical patients affected by PAA, potentially influencing the therapeutic choices in terms of timing and type of surgery. Moreover, patients with an NLR >5 showed a higher risk of long-term adverse events and should be subjected to a more tailored follow-up.

## Figures and Tables

**Table 1:** Clinical and demographic characteristics of all patients included in present series.

	Total Population 97 PAA	LOW-NLR 87 PAA	HIGH-NLR 10 PAA	*p* Value
NLR (mean ± SD)	2.94 ± 2.4	2.32 ± 1.03	8.2 ± 4.4	
Male sex (n;%)	94; 96.9	85; 97.7	9; 90	0.18
Age year (mean ± SD)	69.3 ± 9.3	68.7 ± 8.8	74.5 ± 12.4	0.63
Elective treatment (n;%)	67; 69.1	64; 73.5	3; 30	0.004
Smokers, former or active (n;%)	73; 75.2	64; 73.56	9; 90	0.25
Hypertension (n;%)	75; 77.3	66; 75.8	9; 90	0.31
Dislipidemia (n;%)	48; 49.5	46; 52.8	2; 20	0.04
Diabetes (n;%)	18; 18.5	17; 19.5	1; 10	0.46
CAD (n;%)	34; 35	32; 36.8	2; 20	0.29
COPD (n;%)	27; 27.8	25; 28.7	2; 20	0.55
CKD (n;%)	7; 7.2	6; 6.9	1; 10	0.71
Bilateral PAA (n;%)	46; 47.4	44; 50.6	2; 20	0.06
Cuncurrent AAA (n;%)	30; 30.9	26; 29.9	4; 40	0.51

NLR: neutrophil-to-lymphocyte ratio, SD: standard deviation, CAD: coronary artery disease, COPD: chronic obstructive pulmonary disease, CKD: chronic kidney disease, and PAA: popliteal artery aneurysm.

**Table 2:** Indications and technical details for all the reinterventions performed in present series.

	Reason for Reintervention	Performed Reintervention
In hospital (4)	Bleeding Bypass occlusion (3)	Bleeding control surgery; Embolectomy plus stenting; Embolectomy plus distal anastomosis revision; Embolectomy + new fem-pop bypass (Dacron 6 mm)
30-day (8)	Bleeding (3) Endograft occlusion Ischemic skin necrosis Bypass occlusion (3)	Bleeding control surgery; Ligation of patent collateral vein of an in situ GSV bypass; Embolectomy; Fibrinolysis ± mechanical thrombectomy

GSV: great saphenous vein.

**Table 3:** Demographic parameters compared to NLR values as potential predictors of freedom from major amputation.

	HR	95% CI	*p* Value
NLR > 5	0.033	0.002–0.652	0.025
Age > 75 y	2.702	0.197–38.96	0.456
Smokers, former or actual	0.151	0.01–2.378	0.179
Hypertension	2.651	0.391–17.988	0.318
Dyslipidemia	0.929	0.148–5.819	0.937
Diabetes	0.554	0.026–11.821	0.705
CAD	4.484	0.227–88.573	0.324
COPD	1.063	0.63–17.977	0.966

NLR: neutrophil-to-lymphocyte ratio, HR: hazard ratio, CI: confidence interval, CAD: coronary artery disease, COPD: chronic obstructive pulmonary disease, and CKD: chronic kidney disease.

**Table 4:** Demographic parameters compared to NLR values as potential predictors of primary patency.

	HR	95% CI	*p* Value
NLR > 5	3.101	0.715–13.441	0.130
Age > 75 y	1.810	0.522–6.283	0.350
Sex	0.693	0.093–9.974	0.975
Smokers, former or actual	0.458	0.152–1.381	0.165
Hypertension	2.503	0.933–6.712	0.068
Dyslipidemia	0.872	0.334–2.278	0.780
Diabetes	2.397	0.385–14.915	0.349
CAD	2.244	0.591–8.521	0.235
CKD	0.601	0.109–3.314	0.559
BPCO	0.405	0.129–1.276	0.123

NLR: neutrophil-to-lymphocyte ratio, HR: hazard ratio, CI: confidence interval, CAD: coronary artery disease, COPD: chronic obstructive pulmonary disease, and CKD: chronic kidney disease.

**Table 5:** Demographic parameters compared to NLR values as potential predictors of reintervention.

	HR	95% CI	*p* Value
NLR > 5	0.986	0.112–8.663	0.990
Age > 75 y	1.661	0.474- 5.824	0.428
Sex	1.140	0.112–11.651	0.912
Smokers, former or actual	0.378	0.114–1.250	0.111
Hypertension	2.369	0.850–6.601	0.099
Dyslipidemia	0.977	0.353–2.700	0.964
Diabetes	0.820	0.177–3.797	0.800
CAD	3.481	0.799–15.162	0.097
CKD	0.533	0.098–2.897	0.467
BPCO	0.516	0.159–1.668	0.269

NLR: neutrophil-to-lymphocyte ratio, HR: hazard ratio, CI: confidence interval, CAD: coronary artery disease, COPD: chronic obstructive pulmonary disease, and CKD: chronic kidney disease.

**Table 6:** Demographic parameters compared to NLR values as potential predictors of mortality.

	HR	95% CI	*p* Value
NLR > 5	0.907	0.243–3.388	0.885
Age > 75 y	1.371	0.428–4.394	0.596
Smokers, former or actual	1.454	0.619–3.416	0.391
Hypertension	0.828	0.305–2.250	0.711
Dyslipidemia	0.620	0.251–1.535	0.302
Diabetes	0.596	0.219–1.622	0.311
CAD	1.506	0.587–3.862	0.394
CKD	0.569	0.125–2.601	0.467
BPCO	1.337	0.461–3.880	0.593

NLR: neutrophil-to-lymphocyte ratio, HR: hazard ratio, CI: confidence interval, CAD: coronary artery disease, COPD: chronic obstructive pulmonary disease, and CKD: chronic kidney disease.

## Data Availability

The original contributions presented in this study are included in the article. Further inquiries can be directed to the corresponding author.
